# A systematic review and meta-analysis of the effects of temperature on the development and survival of the *Aedes* mosquito

**DOI:** 10.3389/fpubh.2022.1074028

**Published:** 2022-12-19

**Authors:** Nik Muhammad Hanif Nik Abdull Halim, Nazri Che Dom, Rahmat Dapari, Hasber Salim, Nopadol Precha

**Affiliations:** ^1^Centre of Environmental Health & Safety, Faculty of Health Sciences, Universiti Teknologi MARA (UiTM), UITM Cawangan Selangor, Puncak Alam, Malaysia; ^2^Setiu District Health Office, Setiu, Malaysia; ^3^Integrated Mosquito Research Group (I-MeRGe), Universiti Teknologi MARA (UiTM), UITM Cawangan Selangor, Puncak Alam, Malaysia; ^4^Institute for Biodiversity and Sustainable Development (IBSD), Universiti Teknologi MARA, Shah Alam, Malaysia; ^5^Department of Community Health, Faculty of Medicine and Health Sciences, Universiti Putra Malaysia, Serdang, Malaysia; ^6^School of Biological Sciences, Universiti Sains Malaysia, Penang, Malaysia; ^7^Department of Environmental Health and Technology, School of Public Health, Walailak University, Nakhon Si Thammarat, Thailand

**Keywords:** *Aedes*, dengue (DENV), meta-analysis, temperature, effect

## Abstract

**Introduction:**

The *Aedes* mosquito species, which are the vectors for the transmission of the dengue virus (DENV) to humans, are becoming increasingly susceptible to the formidable effects of influential factors, especially temperature. However, there are still very few studies that have systematically reviewed the existing literature. Hence, in the present study, a systematic literature review and meta-analysis was conducted into the effects of temperature on dengue vectors.

**Method:**

Several research methodologies were incorporated into the current study, and a review was carried out using PRISMA as a guide. The publications for this study were chosen from two prominent databases, Scopus and Web of Science. All of the studies were assessed, reviewed, and evaluated independently by two reviewers. The meta-analysis tool, Review Manager (RevMan Copenhagen Version 5.4.1), was used to record the extracted data for the meta-analysis. Moran's *I*^2^ and a funnel plot were utilized to measure heterogeneity, and publication bias was investigated. A 95% confidence interval (CI) and overall risk difference (RD) were estimated using a random-effects model.

**Result and discussion:**

As a consequence of the search efforts, a total of 46 articles were selected for inclusion in the systematic review and meta-analysis. This review was divided into five major themes, based on a thematic analysis: (i) hatching rate, (ii) development time, (iii) longevity, (iv) survival rate, and (v) wing morphology. In addition, the development time, survival rate, and wing morphology revealed significantly higher risk differences between the maximum and minimum temperatures (RD: 0.26, 95% CI: 0.16, 0.36; *p* = < 0.00001; RD: 0.10, 95% CI: 0.05, 0.14; *p* < 0.0001; and RD: 0.07, 95% CI: 0.02, 0.12; *p* = 0.006, respectively). This study makes several substantial contributions to the body of knowledge and to practical applications. Finally, a number of recommendations are made at the conclusion of this research for the future reference of researchers.

## Introduction

Dengue fever (DF) is an arboviral disease that can spread rapidly through regions and nations, particularly in tropical areas, to cause mild to severe symptoms, including high fever, myalgia, arthralgia, malaise, and pain behind the eyes (1). Based on a 30-fold increase in the incidence of DF over the past five decades, dengue fever is now the most widespread re-emerging mosquito-borne disease and a national hazard. Khetarpal and Khanna (2) discovered that DF is being distributed and is expanding to 128 countries, putting 3.97 billion people at risk annually. Packierisamy et al. (3) initially identified continuous endemic instances of dengue fever (DF) that were harmful to public health in Malaysia in 1902 on Penang Island, while dengue haemorrhagic fever (DHF) was first documented by Kumarasamy (4) in 1962. In addition, since the late 1980s, when the incidence of dengue cases rose sharply, endemic outbreaks of emerging and re-emerging dengue fever (DF) cases have persisted in Malaysia despite disease prevention and control efforts by the government, non-governmental organizations (NGO), and the public.

With the *Aedes* species as the biological vector of DF, there are a number of influential factors that contribute to the incidence of dengue, such as vector density, vector survivability, socioeconomic factors, rainfall distribution, and environmental factors. Rao et al. (1) summarized these into four major factors: epidemiological, virus risk, abiotic risk, and human risk factors. Socioeconomic factors influence the level of dengue awareness of a population (5), whereas Maamor et al. (6) demonstrated that the survival of vectors as a result of their diet and the availability of breeding water may cause a DF outbreak within a 200-m radius of a residential area. In addition, Reinhold et al. (7) hypothesized that an increase in environmental temperature alters the gonotrophic cycle of mosquitoes, resulting in an increase in the rate of disease transmission over a shorter period of time. In light of the fact that everything is directly or indirectly tied to a DF epidemic, which may pose a severe public health risk, preventing, and controlling DF is a challenge.

The ecology of mosquitos is occasionally investigated as they are vectors for DF. Thus far, in Malaysia, studies have confirmed that the ecology of mosquitoes is influenced by rapid unplanned urbanization with defective water supply and solid waste management (8), while artificial light affects the reproductive activities of the Aedes mosquito by lengthening its gonotrophic cycle (9). Other essential elements of the ecology of the mosquito, as referred to by Ahmad et al. (10), include a distinct preference for human habitats and skip oviposition behavior, as well as environmental factors such as heavy rainfall and high temperatures, which are reported to have a strong correlation with the breeding of vector mosquitos and the size of the *Aedes* population (11).

When the mosquito ecology is stratified within a narrow scope, it has been demonstrated that temperature is a crucial determinant influencing the biology of mosquito vectors such as larval development and survivability (9). Shahrudin et al. (12) stated that when exposed to higher constant temperatures, *Aedes albopictus* embryos develop swiftly and their mortality increases, as verified by Rozilawati et al. (13), resulting in a decrease in mosquito fecundity and body mass. Meanwhile, the research by Wang et al. (14) on the impact of a large diurnal temperature range (DTR) on the development of the mosquito population revealed findings that contradicted the findings of many studies that linked temperature with the spread of DF. Despite the contradictory results from studies on temperature and the *Aedes* species, Yeap et al. (15) suggested that normal thermal exposure in field temperatures modifies the morphometric features of the female *Aedes aegypti*. In general, the wings of Aedes mosquitoes are shorter at higher temperatures than at lower temperatures (16), and smaller adults are associated with faster larval development at higher temperatures (17). As a result, smaller Aedes females are better able to select a breeding site and increase the rate of dengue virus infection and spread (18).

Unpredictable weather patterns are becoming more common as a result of climate change. As a result, Brady et al. (19) built a modeling framework on DF cases in relation to the factor of temperature. However, the results showed that the persistence and competence of the *A. aegypti* and *A. albopictus* with regard to the transmission of the dengue virus are not affected by temperature, but rather by the geographical area and type of species. However, temperature is often considered as the main driver that contributes to the development and survival of the *Aedes* species, and it is necessary to explain adequately the variation in temperature that affects these vectors. Temperature range considered suitable for mosquito development, and variations that can positively or negatively affect this development. To test this hypothesis, empirical data is needed with regard to the development and survival of the mosquitos in response to various ranges of temperature. It may be challenging to collect data on such a wide scale in a single experiment or study. However, a meta-analysis of a collection of published estimates of effects with regard to diverse temperature ranges might be used to approach such a dataset. In this way, the phenotype of the effect on the *Aedes* species in response to temperature may be evaluated across a broader range of situations and geographical boundaries. With this background, a meta-analysis of data from studies on the *Aedes* species was conducted in order to summarize the effects of different temperature conditions on the hatching, length of development, survival rate, longevity, and wing morphology of the *Aedes* mosquito and to determine how they affect each other.

## Materials and methods

### Literature search and study selection

This section discusses the method that was used to retrieve articles in relation to the effects of temperature on dengue vectors. This method was employed to run the systematic review on two resources (Scopus and Web of Science) with eligibility and exclusion criteria, while the steps in the review process consisted of identification, screening, and eligibility as well as data abstraction and analysis.

**Figure 1:**
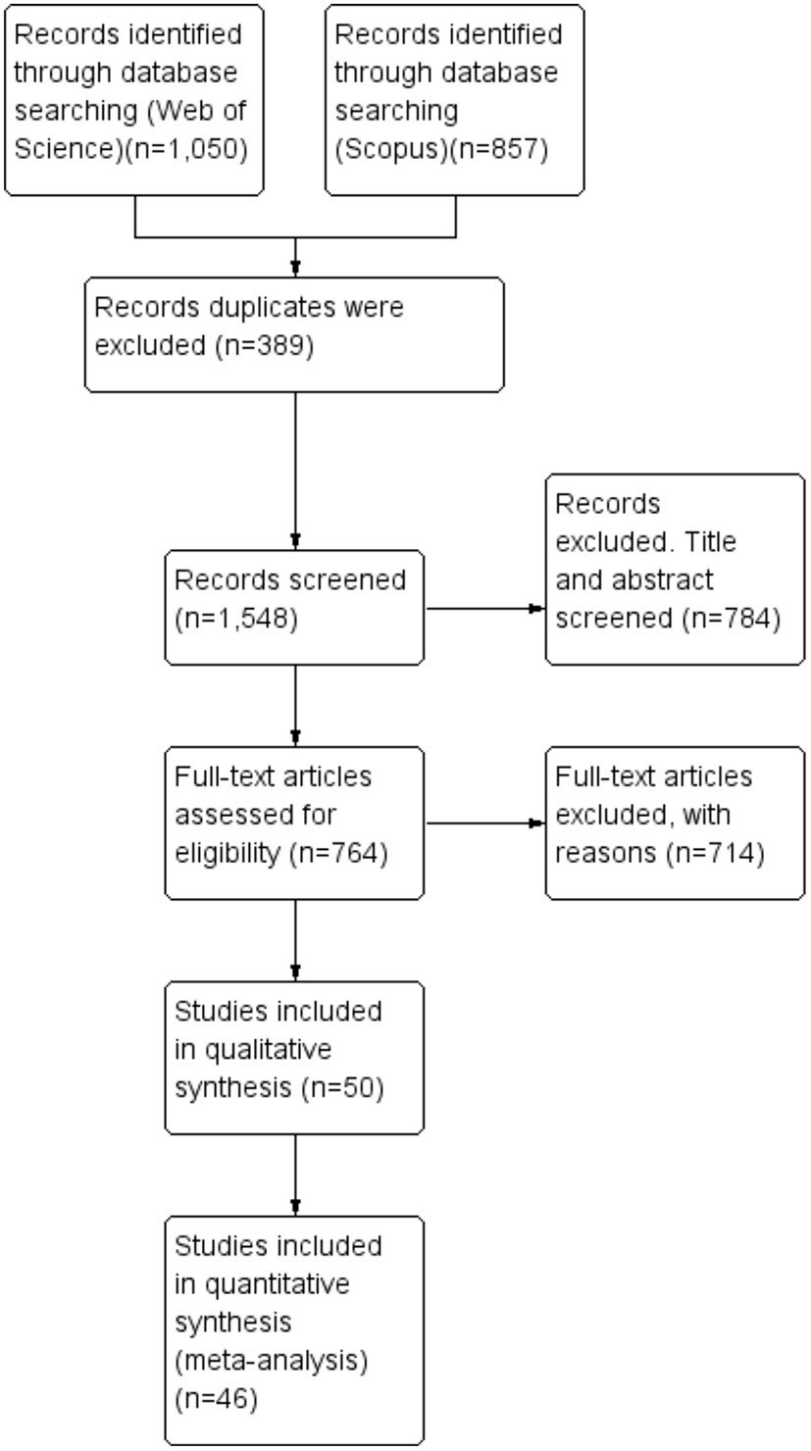
Flow diagram of the selection of studies for the review process.

This review was guided by the method known as PRISMA or *Preferred Reporting Items for Systematic Review and Meta-Analysis*. It is a published standard protocol for conducting a systematic literature review and meta-analysis. In general, this publication standard is used as a guide in the evaluation and examination of the quality and purity of a review with the necessary and related information provided. Moreover, PRISMA highlights a report of the review, evaluating randomized trials that can also be the basis for other types of research in reporting systematic reviews (20). On the other hand, PRISMA is also suitable for the field of environmental management since it clearly emphasizes on research questions with regard to the need for systematic reviews, and it is also a fact that PRISMA has always been used in medical studies, especially for public health, as it can be a tool to identify the inclusion and exclusion criteria for a particular study (21). Furthermore, PRISMA also extensively examines databases if a literature review is unavailable at a particular time in order to allow searching for the accurate term in relation to the effects of climatic and environmental factors on dengue vectors. In addition, the use of PRISMA enhances the coded information for involvement in future environmental management services. The results of this review were presented following the PRISMA flow diagram as in Figure 1. The protocol for this systematic review and meta-analysis was not registered.

### Resources

The method for the present review was conducted using two main databases; (i) Scopus and (ii) Web of Science (WoS). These two databases were used as they are the leaders in systematic reviews among all the databases, and they mostly cover more than 256 fields of study or subject categories, including environmental science. The WoS database consists specifically of more than 33,000 journals and 256 subject categories related to environmental studies, social science, and interdisciplinary social science and development planning issues. It also covers over 100 years of comprehensive back files and citation data established by Clarivate Analysis and ranks them by three components, namely papers, citations, and citations per paper. The second database that was used in this review was Scopus, which is recognized as one of the largest abstract and citation databases of peer-reviewed articles with more than 22,800 journals from 5,000 publishers across the world covering various subject categories, for instance, social science, environmental science and biological science.

### Formulation of research question

In this review, the formulation of a suitable research question was developed using PICO as an assistant tool. PICO is comprised of three components, namely Population or Problem, Interest and Context. Three aspects were covered in this review based on these three components, namely effect (problem), temperature (interest) and dengue vectors (context), which were then used as a guide in the development of the main research question—What are the effects of temperature on the development and survival of the *Aedes* mosquito?

### Systematic searching strategies/systematic review process

Three main steps were involved in the systematic searching process to select the relevant articles for this review, namely identification, screening and eligibility. The first stage of identification involved identifying variations of the main keywords. This was followed by the process of searching for any synonyms and related terms based on dictionaries, thesauri, encyclopedia, and relying on past studies and keywords from experts. This process was carried out to obtain more options so as to search for more articles for the review. Next, the existing keywords and search strings were enriched based on Boolean operators, phrase searching, truncations, wild cards and filed code functions on the main databases of Scopus and Web of Science (WoS), which were developed in January 2022 after all the related keywords had been fully accessed.

This review screened all the selected articles retrieved from the identification process by choosing the criteria for the selected articles. This was done automatically based on the sorting function in the database. The first step in the screening process was to remove duplicate articles. During this process, some of the articles were excluded, while the rest were screened for the second step based on several inclusion and exclusion criteria. In this review, the first criterion considered the type of publication, which included only articles with complete empirical data that had been published in a journal as a primary source rather than published in the form of a systematic review, meta-analysis, chapters in books and conference proceedings in order to ensure the quality of the review. Moreover, this review only focused on articles that were published in English to avoid any confusion in understanding. In addition, a timeline of between 2012 to 2021 was included as one of the inclusion criteria. This timeline of 10 years back was chosen since search should be as wide as possible to maximize the likelihood of capturing all relevant data and the publication related to Aedes mosquitoes rise in this period of time. As it was impossible to conduct a search of all the published articles, thus, a fixed period was determined to enable the review to be carried out (22).

All the remaining articles were prepared for the third step, namely, eligibility. During this process, the articles were presented to two experts for a quality assessment to ensure that the contents of the articles were of good quality. Most importantly, the titles, abstracts, and the main contents of the articles were examined clearly to ensure they fulfilled and matched all the inclusion criteria so as to accomplish the objective of the review. Consequently, all the remaining articles selected from the last stage were further analyzed by means of a qualitative analysis.

### Quality appraisal/risk of bias assessment

In order to ensure the quality of the contents, the remaining articles from the previous process were presented to two experts for a quality assessment. The experts ranked the articles according to three indicators, namely, low, moderate and high bias. Consequently, only low and moderately biased articles were selected for the review. To determine the quality of the articles, both the experts ranked their quality based on the methodology of the article. Both the authors had to mutually agree that at least a moderate quality article was included in the review. Any argument that came up during the assessment was discussed among them before a decision was made in terms of the inclusion or exclusion criteria for further analysis.

**Figure 2:**
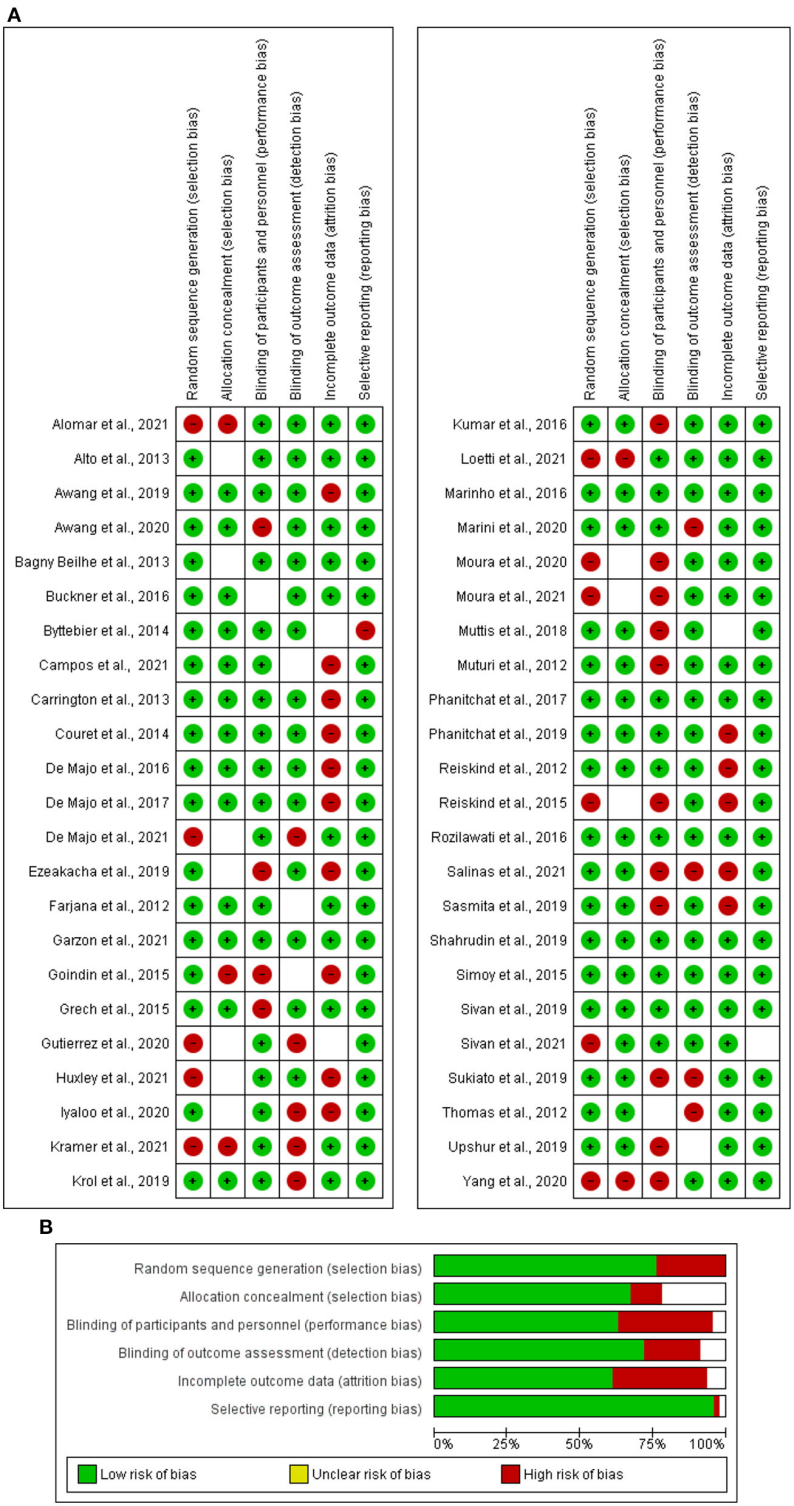
**(A)** Summary of risk of bias based on traffic light plot. **(B)** Graph of risk of bias.

The Joanna Briggs Institute Tool (JBI) tool 29 criteria was used to assess the quality of the articles chosen for this study. There were eight questions on the checklist, namely: (A) Were the criteria for inclusion in the sample clearly defined? (B) Were the study subjects and setting described in detail? (C) Was the exposure measured in a valid and reliable way? (D) Were objective standard criteria used for measuring the conditions? (E) Were confounding factors identified? (F) Were strategies to deal with confounding factors stated? (G) Were the outcomes measured in a valid and reliable way? and (H) Was an appropriate statistical analysis used? The questions were checked against the 46 articles, and answers were given based on the indication of (+) for “Yes” or (–) for “No.” The risk of bias in the selected studies was assessed using robvis (visualization tools). Two authors separately evaluated the possibility of bias in each publication. The checklist consisted of five questions. The risk of bias was classified as “low risk,” “high risk,” or “unclear risk” (Figure 2A). A summary bar chart was constructed using the same dataset (Figure 2B).

### Data abstraction

This review relied on an integrative review, a technique that requires the analysis and synthesis of diverse research designs. This means that quantitative, qualitative and mixed-mode approaches were included in this review. A good approach would be to analyse the integrative data using qualitative or mixed mode techniques so as to enable a comprehensive comparison to be made across the data sources by the authors (23). In this review, all the articles selected in the abstract, results and discussion sections were read thoroughly. The data abstraction process was conducted based on the research question. The data obtained from the reviewed studies were those that were able to answer the research question, and they were, then, summarized in the form of a table. Next, a thematic analysis was performed to determine the themes and sub-themes in order to present the patterns, counting and clustering, and to identify the similarities and associations existing in the summarized data (24). A thematic analysis was considered since it is the most applicable for synthesizing integrative or mixed research designs (25). This kind of descriptive study reduces the flexible mode data that have been merged with other techniques of data analysis (26).

The initial step in the thematic analysis was to generate the themes, and to identify the patterns that appeared in the summarized data of all reviewed articles. Any similarities between the reviewed data were pooled together under one theme. Then, the themes were scrutinized once again and other sub-themes were identified. Next, the process was continued by reviewing the efficiency of the themes. In this step, all the main themes and sub-themes that were generated were scrutinized to ensure the effectiveness and accuracy of the data representation. During this process, some themes might be excluded due to the inclusion criteria that were determined for this study. The next stage was choosing the proper names for the main themes as well as their sub-themes. Within the theme development process, this technique included the involvement of corresponding authors and co-authors in identifying the most relevant themes related to the objective of this review. The group of authors discussed any ideas, inconsistencies, similarities and associations with the data interpretation until a point of agreement was reached on the development of the themes. Following this, the developed themes and their sub-themes were presented to two panel experts, both of whom were experts in qualitative studies. These experts were asked to classify all the themes developed instinctively and decide whether the themes and sub-themes were relevant and appropriate for the review results.

### Statistical analysis

The remaining articles were assessed and evaluated. The focus of the research efforts was on specific studies that provided answers to the posed issues. The Review Manager (RevMan Copenhagen Version 5.4.1: The Nordic Cochrane Centre, 2012), which is a meta-analysis tool, was used to record the data extracted from the 46 papers in order to conduct a meta-analysis. Before the data could be submitted into RevMan, a lot of work had to be done. A considerable amount of time was required for the literature search. Since a meta-analysis relies on data from publications, certain data must be transformed. The outcomes of the analysis were represented as forest plots. Sub-group analyses were utilized to stratify the studies that employed distinct or a combination of interventions. Cochrane's *Q* (Chi-square) and Moran's *I*^2^ (Inconsistency) tests were used to assess heterogeneity. If the *p-*value was < 0.05, then, heterogeneity was considered to be statistically significant, while *I*^2^ values of 25, 50, and 75% were deemed to reflect low, moderate, and high levels of heterogeneity, respectively.

## Results

### General findings and background of selected articles

More specifically, it should be noted that of the 46 studies that were included, 11 studies were geographically located in the United States, 10 in Argentina, five in Malaysia, three each in Brazil and India, two each in France and Thailand, and one each in China, Germany, Italy, Japan, Mauritius, Mexico, Nepal, South Africa, Taiwan, and the United Kingdom. As mentioned in this study, a combination of experimental and field study designs was mostly selected to determine the goal of the study in relation to dengue vector studies compared to the application of either a field or experimental study design. Furthermore, 21 studies were experimental studies, while only two were field studies. The remaining 23 studies were a combination of both experimental and field studies. It was not the goal of this study to present a list of the best study designs to assess the studies related to dengue vectors in terms of the robustness of the methods. However, on considering the types of research designs that were identified during the analysis, it was noted that there was an interesting balance between experimental and field studies. On the other hand, the results also represented the key elements of the study with regard to the mosquito strains that were selected for further analysis, where 29 articles reported on the *A. aegypti*, nine articles examined the *A. albopictus*, and seven articles considered both the *A. aegypti* and *A. albopictus*, while 1 article studied a combination of the *A. aegypti* and Culex spp., respectively. Table 1 summarizes the findings, research period, location, type of study, study design, and variables observed in the evaluated studies on the effects of climatic and environmental conditions on dengue vectors. Based on the thematic analysis, five themes were developed regarding the effects of temperature on dengue vectors, namely, the hatching rate (%), development time (days), survival rate (%), longevity (days), and wing morphology (mm).

**Table 1:** Summary of the articles related to effect of temperature on Aedes mosquito.

**ID**	**Author**	**Study location**	**Mosquito strain**		**Study design**		**Effect on dengue vectors**							**Independent variables**						
			**AE**	**AL**	**LW**	**FW**	**Hatching**	**Development time**	**Longevity**	**Survival**	**Wing size**	**Seasonal**	**Diet**	**Container **	**Competence**	**RH**	**Wind speed **	**Density**	**Altitude**	**Light**	**Insecticide**
ID1	Alomar et al. (27)			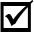			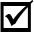			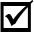										
ID2	Alto and Bettinardi (28)	USA		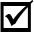	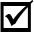			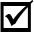	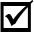	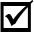	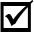										
ID3	Awang et al. (29)	Malaysia		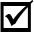	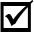			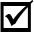													
ID4	Awang and Dom (30)	Malaysia	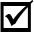	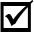	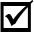	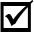		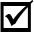	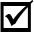	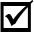	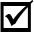			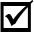		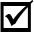	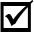				
ID5	Bagny Beilhe et al. (31)	France	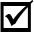	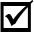	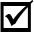		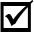				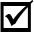		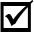		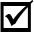			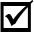			
ID6	Buckner et al. (32)	USA	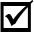	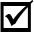	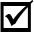			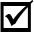		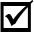			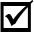								
ID7	Byttebier et al. (33)	Argentina	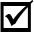		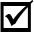		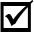							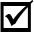							
ID8	Campos et al. (34)	Argentina	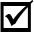		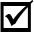	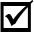	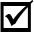	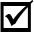			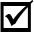										
ID9	Carrington et al. (35)	USA	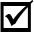		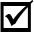	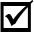			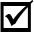	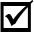	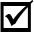										
ID10	Couret et al. (36)	USA	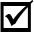		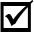		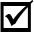	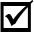					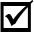					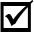			
ID11	De Majo et al. (37)	Argentina	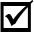		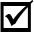	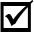	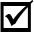	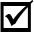		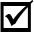											
ID12	De Majo et al. (38)	Argentina	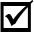		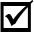	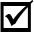	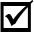	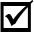		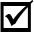											
ID13	De Majo et al. (39)	Argentina	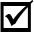		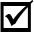	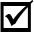		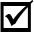	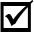	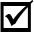	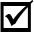										
ID14	Ezeakacha and Yee (40)	USA		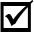	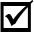			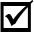							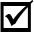						
ID15	Farjana et al. (41)	Japan	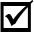	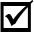	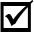			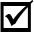		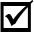	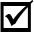		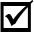		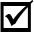						
ID16	Garzón et al. (42)	Argentina	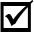	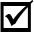	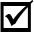		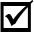				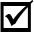										
ID17	Goindin et al. (43)	France	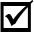		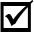			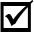													
ID18	Grech et al. (44)	Argentina	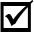		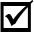	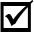		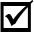		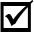											
ID19	Gutiérrez e al. (45)	USA	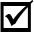		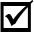	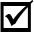		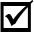		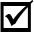	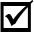										
ID20	Huxley et al. (46)	UK	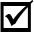		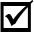			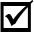					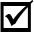								
ID21	Iyaloo et al. (47)	Mauritius		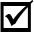		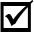			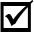												
ID22	Kramer et al. (48)	Nepal	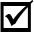	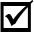	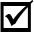	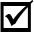		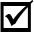	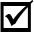		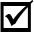								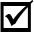		
ID23	Krol et al. (49)	S. Africa	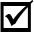			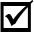				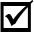				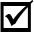	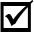						
ID24	Kumar et al. (50)	India	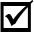		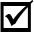			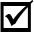						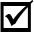							
ID25	Loetti et al. (16)	Argentina	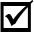		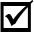			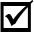	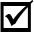	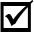	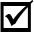									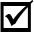	
ID26	Marinho et al. (51)	Brazil	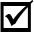		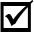	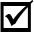		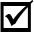	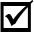												
ID27	Marini et al. (52)	Italy		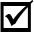	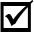			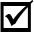	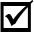										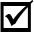		
ID28	Moura et al. (17)	Brazil	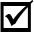		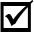				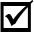		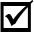										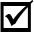
ID29	Moura et al. (18)	Brazil	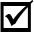		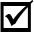			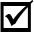													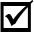
ID30	Muttis et al. (53)	Argentina	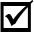		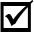	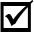	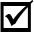	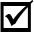	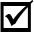												
ID31	Muturi et al. (54)	USA	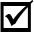		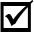			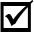		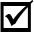	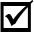				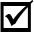			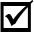			
ID32	Phanitchat et al. (55)	Thailand		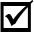	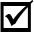	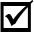	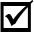	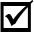													
ID33	Phanitchat et al. (56)	Thailand		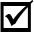				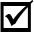	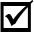	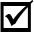	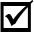										
ID34	Reiskind and Zarrabi (57)	USA		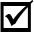	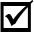	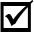		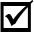		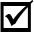			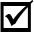								
ID35	Reiskind and Janairo (58)	USA	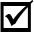		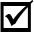	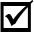		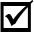		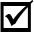			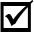	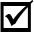							
ID36	Rozilawati et al. (13)	Malaysia	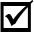	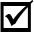	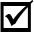	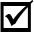		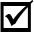	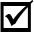		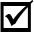										
ID37	Salinas et al. (59)	Mexico	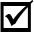	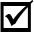	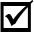	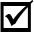				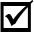											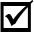
ID38	Sasmita et al. (60)	Taiwan	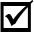		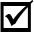			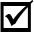	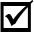	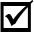	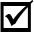		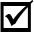								
ID39	Shahrudin et al. (12)	Malaysia	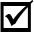		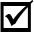			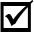	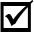												
ID40	Simoy et al. (61)	Argentina	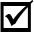		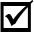			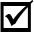													
ID41	Sivan et al. (62)	India	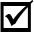		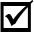	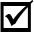	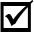			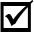											
ID42	Sivan et al. (63)	India	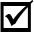		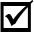	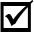	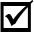			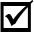											
ID43	Sukiato et al. (64)	Malaysia	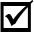		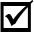		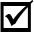	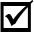		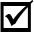	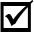									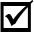	
ID44	Thomas et al. (65)	Germany	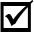	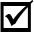	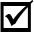	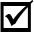				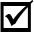											
ID45	Upshur et al. (66)	USA	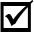		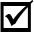			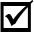	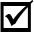		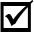		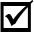								
ID46	Yang et al. (67)	China	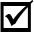		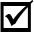	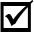	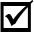		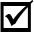												

AE, Aedes aegypti; AL, Aedes albopictus; LW, experimental studies; FW, field works; RH, relative humidity.

### Effects of temperature on *Aedes* mosquitoes

#### Hatching rate

**Figure 3:**
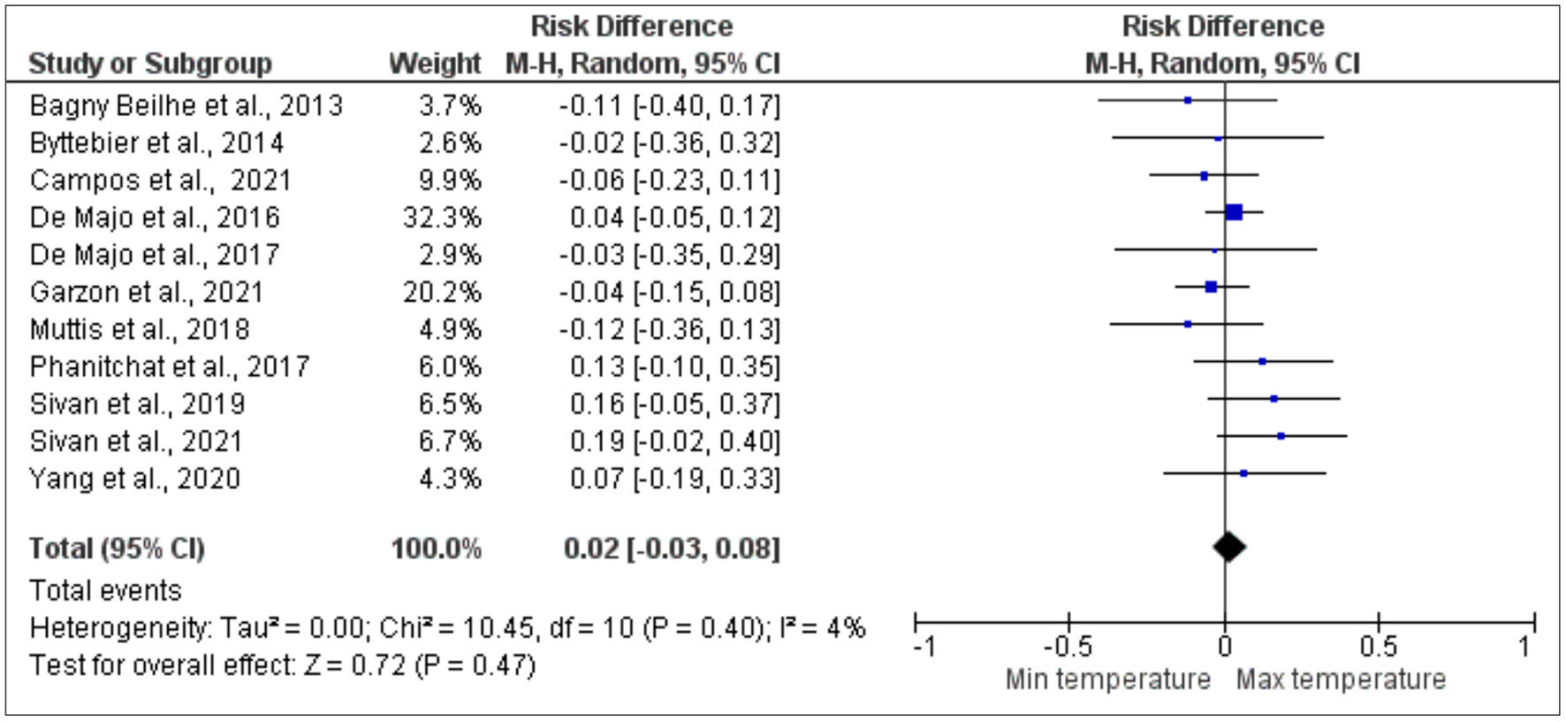
Forest plot of comparison of the effect of minimum temperature vs. maximum temperature on the hatching rate of Aedes mosquito.

Out of the 46 studies that were analyzed, 11 studies, which gave information about the effect of the hatching rate on dengue vectors using the risk difference between the maximum and minimum temperatures, were included in one meta-analysis (Figure 3). The overall estimate of heterogeneity across the studies was also very low, with *I*^2^ = 4%, and this was probably due to the small number of studies that were included for further analysis. The hatching rate showed a higher risk difference between the maximum and minimum temperatures without any significant effect on the dengue vectors. More specifically, based on the analysis, the studies by Sivan et al. (63), Sivan et al. (62), Phanitchat et al. (55), Yang et al. (67), and De Majo et al. (38) showed no significant higher risk difference between the maximum and minimum. On the other hand, studies by Bagny Beilhe et al. (31), Byttebier et al. (33), Campos et al. (34), De Majo et al. (37), Garzón et al. (42), and Muttis et al. (53) showed that there was no significant lower risk difference between the maximum and minimum temperatures respectively.

#### Development time

**Figure 4:**
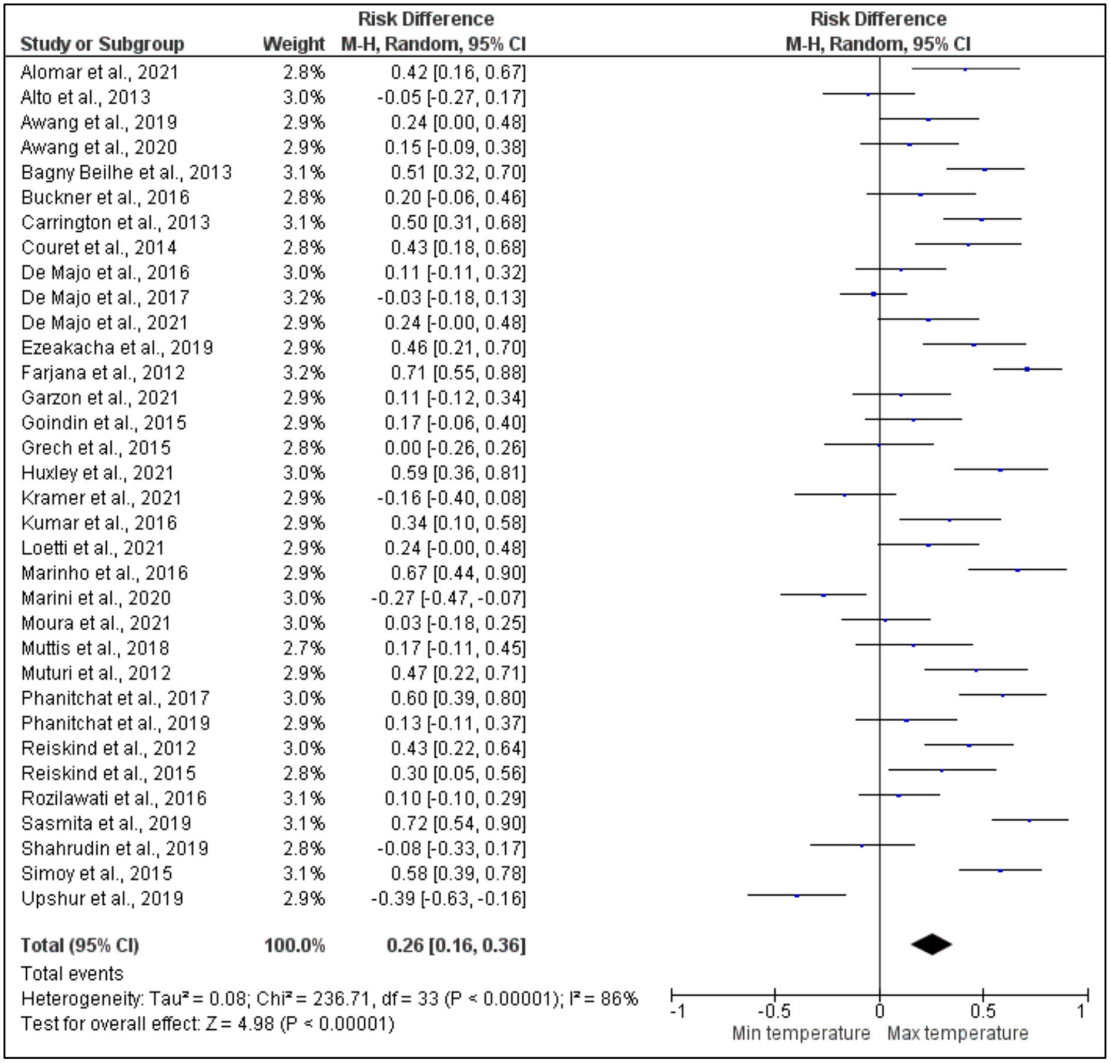
Forest plot of comparison of the effect of minimum temperature vs. maximum temperature on the development period of Aedes mosquito.

After pooling 34 articles for further analysis on the risk difference between the maximum and minimum temperatures that affects dengue vectors, the development time was also revealed to have a higher significant risk difference between the maximum and minimum temperatures with a high heterogeneity across the studies. More specifically, based on the meta-analysis shown in Figure 4, the studies by Phanitchat et al. (55), Bagny Beilhe et al. (31), Alomar et al. (27), Carrington et al. (35), Couret et al. (36), Ezeakacha and Yee (40), Farjana et al. (41), Huxley et al. (46), Kumar et al. (50), Marinho et al. (51), Muturi et al. (54), Reiskind and Zarrabi (57), Sasmita et al. (60), and Simoy et al. (61) showed a higher significant risk difference between the maximum and minimum temperatures, respectively. Conversely, other studies by Marini et al. (52) and Upshur et al. (66) revealed a significant lower risk difference between the maximum and minimum temperatures. However, the analytical results of other studies by Rozilawati et al. (13), Loetti et al. (16), Garzón et al. (42), Awang et al. (29), Awang and Dom (30), Buckner et al. (32), De Majo et al. (39), Goindin et al. (43), Phanitchat et al. (56), and De Majo et al. (38) presented a higher risk difference that was not significant between the maximum and minimum temperatures. Other studies by Shahrudin et al. (12), De Majo et al. (37), Marini et al. (52), Alto and Bettinardi (28), Grech et al. (44), and Kramer et al. (48) showed a lower risk difference that was not significant between the maximum and minimum temperatures.

#### Longevity

**Figure 5:**
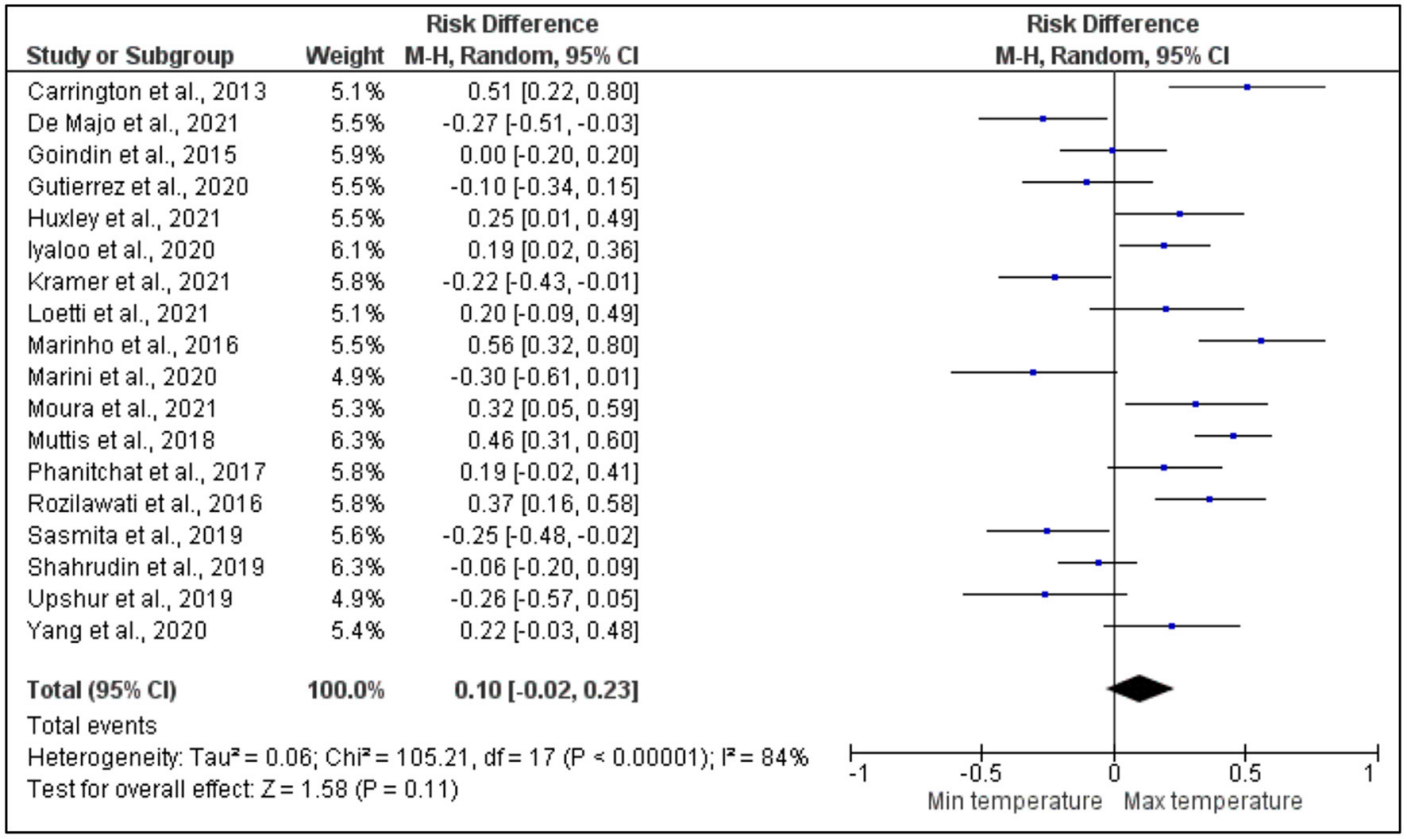
Forest plot of comparison of the effect of minimum temperature vs. maximum temperature on the longevity of Aedes mosquito.

Out of the total number of studies that were analyzed, 18 studies provided information about the effect of the maximum and minimum temperature on the longevity of dengue vectors in the meta-analysis (Figure 5). The longevity showed no significant higher risk difference between the maximum and minimum temperatures that affected the dengue vectors. However, the overall estimate of heterogeneity across the studies was very high which was probably due to the large number of studies that were included in the analysis. More specifically, based on the analysis, studies by Moura et al. (18), Carrington et al. (35), Huxley et al. (46), Iyaloo et al. (47), Marinho et al. (51), Muttis et al. (53) and Rozilawati et al. (13) revealed a significant higher risk difference between the maximum and minimum temperatures. Also, a significant lower risk difference between the maximum and minimum temperatures was revealed in three studies by De Majo et al. (39), Kramer et al. (48), and Sasmita et al. (60). On the other hand, a few studies by Loetti et al. (16), Phanitchat et al. (55), and Yang et al. (67) presented a higher risk difference with no significance between the maximum and minimum temperatures, whereas a few remaining studies by Shahrudin et al. (12), Goindin et al. (43), Gutiérrez e al. (45), Marini et al. (52), and Upshur et al. (66) demonstrated a lower risk difference without any significance between the maximum and minimum temperatures.

#### Survival rate

**Figure 6:**
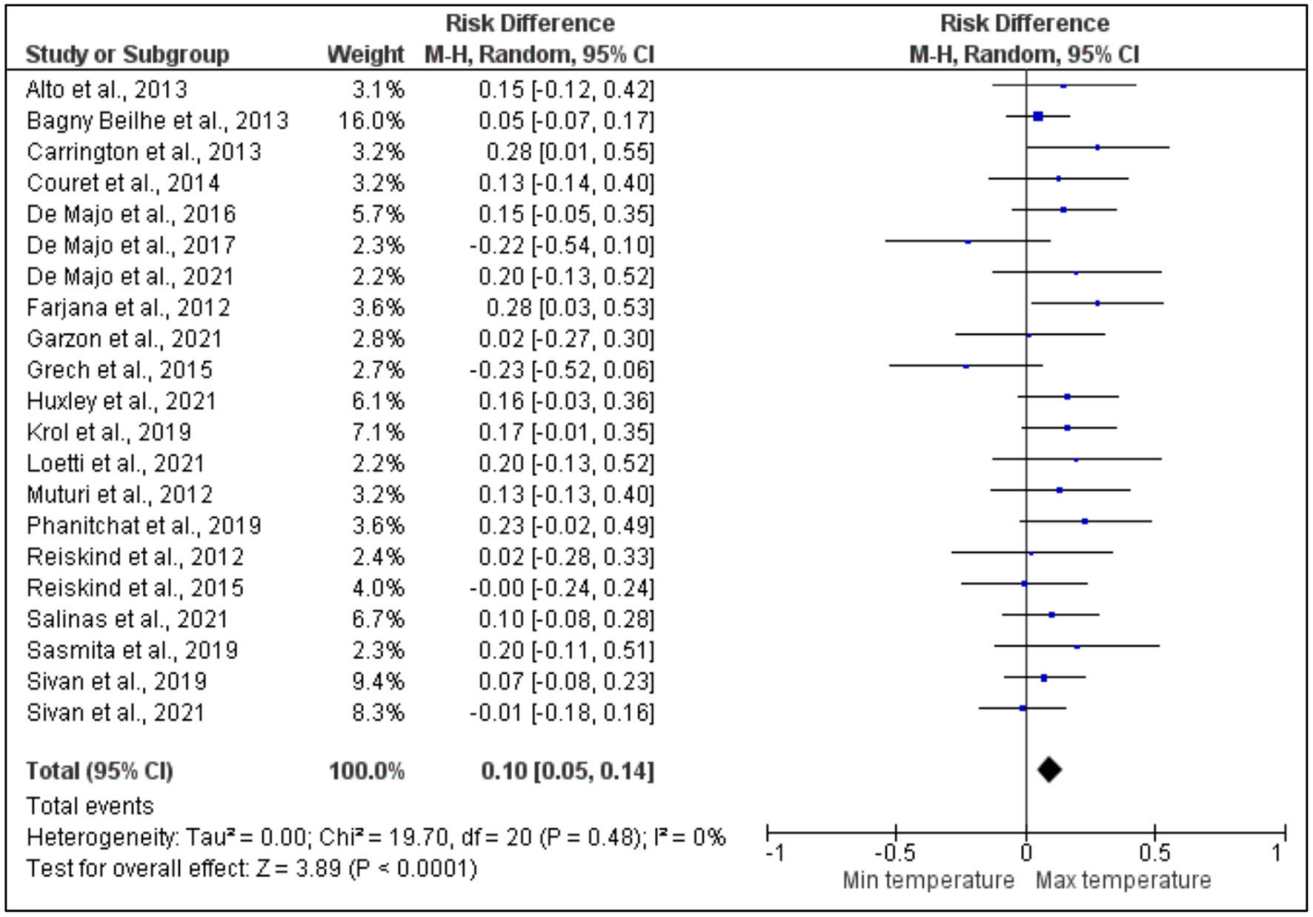
Forest plot of comparison of the effect of minimum temperature vs. maximum temperature on the survival of dengue vectors.

Figure 6 shows the results of the effect of the maximum and minimum temperatures on the survival of dengue vectors after 21 articles were analyzed out of the total number of articles selected for the meta-analysis. Only two studies by Carrington et al. (35) and Farjana et al. (41) showed a significant higher risk difference between the maximum and minimum temperatures. Conversely, studies by Loetti et al. (16), Sivan et al. (63), Bagny Beilhe et al. (31), Garzón et al. (42), Couret et al. (36), Huxley et al. (46), Muturi et al. (54), Reiskind and Zarrabi (57), De Majo et al. (39), Phanitchat et al. (56), Alto and Bettinardi (28), Krol et al. (49), Salinas et al. (59), De Majo et al. (38), and Sasmita et al. (60) revealed a higher risk difference without any significance between the maximum and minimum temperatures. On the other hand, other studies by Sivan et al. (63), De Majo et al. (37), Grech et al. (44), and Reiskind and Janairo (58) demonstrated a lower risk difference without any significance between the maximum and minimum temperatures. The overall estimate of heterogeneity across all the studies was very low which was probably due to the small number studies that were included in the analysis. There was a significant higher risk difference between the maximum and minimum temperatures, thereby indicating their effect on the survival rate of the dengue vectors, while individual researches that had no significant effect of the summary results were excluded.

#### Wing morphology

**Figure 7:**
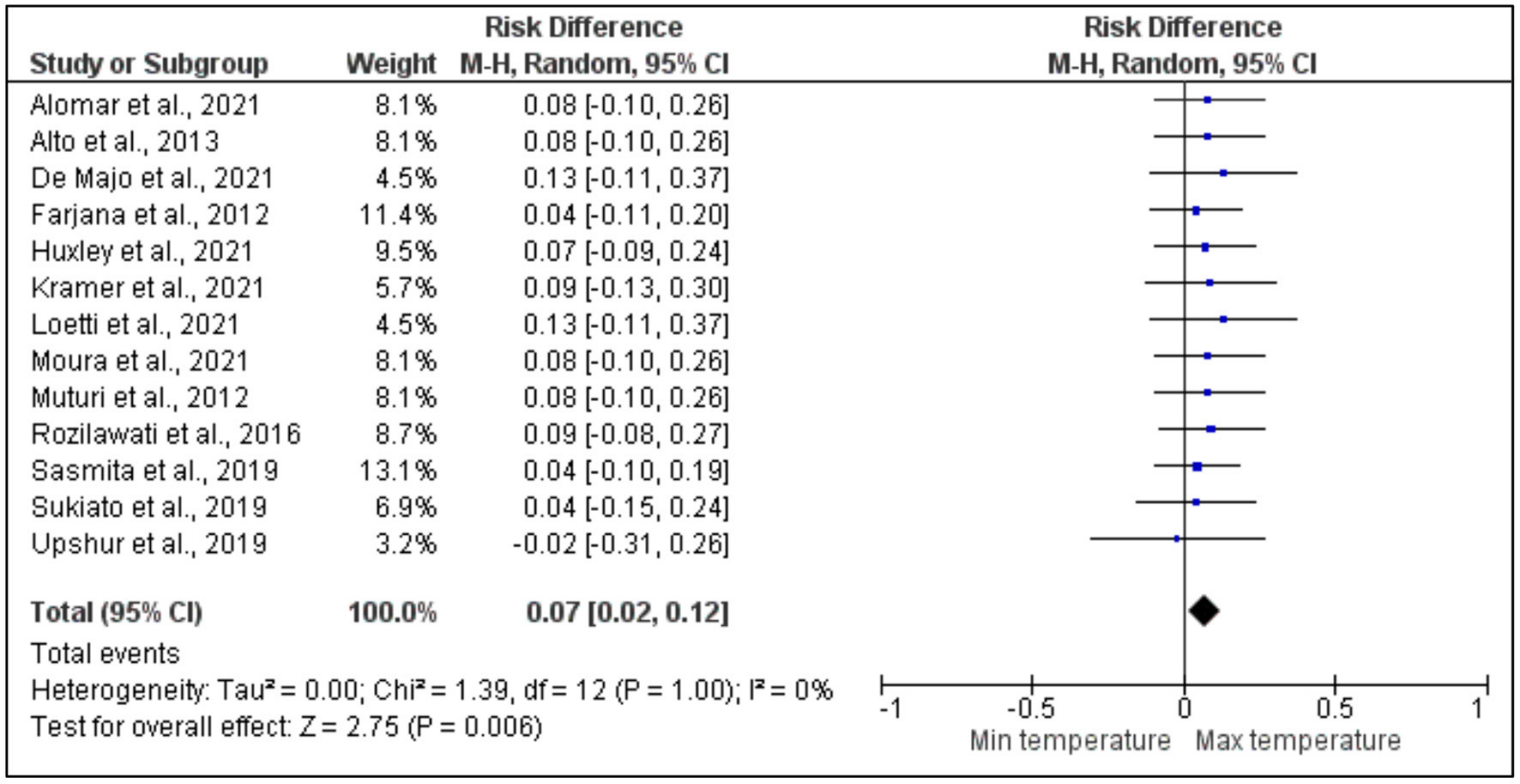
Forest plot of comparison of the effect of minimum temperature vs. maximum temperature on the wing morphology of dengue vectors.

After pooling 13 articles for a further analysis of the effect of the risk difference between the maximum and minimum temperatures on the wing morphology of dengue vectors, it was revealed that there was a higher significant risk difference between the maximum and minimum temperatures. However, there was a low heterogeneity across the studies. More specifically, based on the meta-analysis shown on Figure 7, most of the studies by Rozilawati et al. (13), Loetti et al. (16), Moura et al. (18), Alomar et al. (27), Farjana et al. (41), Huxley et al. (46), Muturi et al. (54), Sasmita et al. (60), De Majo et al. (39), Alto and Bettinardi (28), Kramer et al. (48), and Sukiato et al. (64) revealed a non-significant higher risk difference between the maximum and minimum temperatures, whereas only one study by Upshur et al. (66) showed a non-significant lower risk difference between the maximum and minimum temperatures.

## Discussion

The present study was carried out to systematically analyse the existing literature on the various effects of temperature on dengue vectors, specifically for the *A. aegypti* and *A. albopictus*, and how these factors affect both these species of mosquitoes. A proper review in relation to the effects of temperature on dengue vectors was conducted on 46 articles sourced from two databases. The findings revealed that these factors affect dengue vectors in various ways. Five themes emerged from the thematic analysis that was performed within the scope of this review. Hence, this section presents further discussions on the developed themes.

Based on this review and meta-analysis, temperature is becoming one of the important factors affecting the biology, population continuation and growth of the mosquito. Before a mosquito reaches adulthood, it goes through a few stages in its life cycle. However, both internal and external factors determine whether each stage is successfully completed. Being poikilothermic (cold-blooded) organisms, mosquitoes are sensitive to changes in the ambient temperature, which directly affects their body temperature. Temperature is therefore one of the most significant elements that might affect the insect survival, adult longevity, immature growth, vectorial capacity and other aspects of the life history of the *A. aegypti* and *A. albopictus* (13, 53, 61, 62, 68).

### Hatching rate

Temperature is an essential hatching signal (42, 69). Temperate juvenile *A. aegypti* have successfully acclimated to cooler environments compared to subtropical populations. The eggs of temperate *A. aegypti*, for example, hatch faster at 5°C, 12°C, and 15°C (52). As a result, the spread of the *A. aegypti* to colder regions appears to be possible in the future (8, 48, 63). Another study discovered that during winter the egg hatching rate of the *A. albopictus* at 25°C fell at 11°C (67). Garzón et al. (42) discovered that the percentage of hatching in the *A. aegypti* was higher at 27°C than at 16°C. Most crucially, a temperature threshold of 37°C resulted in a drastically reduced hatching rate for the *A. aegypti*, with the upper limit occurring between 38 and 42°C (70). However, the fact that some larvae still hatched suggests a potential physiological adaptation to high water temperatures of above 39°C (51).

### Development time

As expected, the development time of the *A. aegypti* and *A. albopictus* mosquito is affected by changes in the temperature of the surrounding environment when it exceeds the lower critical development threshold (71). The results showed that changes in the temperature could alter the stage of development starting from the larva stage until the emergence of the adult (30, 36, 64). Estimates of the development time were strongly temperature sensitive and negatively related to temperatures from 35°C until 40°C. Beyond this point, the rate of development slowed and reproduction ceased. Thus, this situation was very harmful to the mosquitoes and increased the mortality rate in both the *A. aegypti* and *A. albopictus* species (34, 41). Larval development was halted, resulting in larval death of 100% as the mosquito larvae then suffered stress as a result of a rise in temperature, which may have caused abnormalities at the cellular level in the larvae. Higher temperatures also stimulated rapid growth, leading to fewer nutrient reserves, which led to molting failure (50). On the same note, the detrimental effect of higher temperatures on culicid vector development (68, 72) suggested that this approached the lethal temperature range for the Aedes mosquito (36, 51).

### Survival rate and longevity

Temperature also had an effect on the lifespan on the *Aedes* adult (17, 60). Temperature extremes of 16°C and 36°C significantly reduced adult longevity and female fertility (51). In terms of sexes, at the most temperatures, the males have shorter lifespan than female. This pattern could be related to the intensity of oviposition, which is both energetically and likely to limit longevity (51). Male longevity decreases mating possibilities, while female longevity decreases gonotrophic cycles (17). According to Muttis et al. (53), the lifetime of the mosquito is more dependent on temperature than on population-related parameters such as density. As a result, it was anticipated that mosquito larvae would live shorter lives at high temperatures. Other than that, it was inferred that the daily temperature dynamics and ambient temperature are significant in an endemic area, which may help with the selection of mosquitoes that can survive at high temperatures, thereby enhancing their ability to transmit diseases (63).

### Wing morphology

Temperature is one of the extrinsic elements that directly influence insect development. Literature suggests that wing and body size are influenced by other environmental conditions such as population density in breeding containers (73, 74) and competence (75). Our results suggest that warmer temperatures also lead to smaller adult sizes and vice versa, a phenomenon known as the temperature-size rule or the “hotter is smaller” rule (76). The estimation of the wing length reveals the mosquito body size that can epidemiologically affect relevant qualities by indicating the responsiveness of the adult mosquito to the development of the juvenile stages. The change in weight and wing length allometry at a particular temperature may be advantageous in a number of different ways. One hypothesis is that the elasticity of wing length and weight may result in lower wing loadings at low temperatures, which could be advantageous at colder operating temperatures due to the increased energy required for a given flying output (57). Measurements of the wing size were performed in order to analyse the impact of variations in the wing size on the ability of the mosquito to select a breeding location and engage in blood feeding in field conditions. The larger size of the female mosquito may indicate that it has a longer flight range than a little mosquito, finds hosts more frequently, feeds successfully on blood, and has superior location skills for oviposition, all of which boost fertility. In light of this, it was determined that the fecundity rate and the capacity to fly to an oviposition site are higher at lower temperatures and decrease as temperatures rise (12).

These findings are necessary for the adoption of dengue outbreak systems and minimization of the adverse effects of the transmission of the dengue virus by its two main vectors, the *A. aegypti* and *A. albopictus*. It is critical to have knowledge of what and how environmental and climatic conditions can affect dengue vectors. However, this study had its limitations, and a number of recommendations have been made that may be beneficial for future research while conducting a meta-analysis, such as the fact that only English-language papers were examined, which might have introduced a linguistic bias into the findings. Then, it was discovered that variations in heterogeneity at several stages of the gonotrophic cycle, including hatchability, development time, survival, longevity, and wing shape, might have been responsible for this heterogeneity. This heterogeneity analysis was impacted by the small sample size, which included the number of studies that were taken into account for the review and meta-analysis. It was demonstrated that the development periods of the *A. aegypti* and *A. albopictus* were shortened, larval and pupal mortality were increased, and hatchability was dramatically reduced as a result of rising temperatures. The *Aedes* population will likely increase in the subtropical and temperate zones, and not necessarily in the tropical areas, due to global warming, and the distribution and seasonal duration will increase. Furthermore, new pandemic hotspots will be appearing. This range for this species has already widened to include greater latitudes and altitudes. As a result, epidemics are more prone to occur and spread over wider areas.

Therefore, information on the life cycle of Aedes mosquitoes at different temperatures was important for planning effective dengue control. The ability of Aedes to survive at different water temperatures showed that the vector is highly adaptable to the ever-changing environment. This study also found that development time and wing morphology accelerate when temperature is increased. This Aedes life data could also serve as an advance warning and is necessary from an epidemiological perspective to identify the pattern of results due to seasonal changes throughout the year. Vector control efforts and population dynamics models will be improved if realistic parameter estimates of mosquito populations are incorporated into future surveillance activities and research projects.

## Data availability statement

The raw data supporting the conclusions of this article will be made available by the authors, without undue reservation.

## Author contributions

NN, RD, and NC wrote the manuscript. HS collected the data and did the analysis. NP proofread and edited the manuscript. All authors contributed to the article and approved the submitted version.
